# Evaluation of acoustic radiation force impulse imaging for the clinicopathological typing of renal fibrosis

**DOI:** 10.3892/etm.2013.1377

**Published:** 2013-10-31

**Authors:** GUANGHE CUI, ZHI YANG, WENXIAO ZHANG, BAOWEI LI, FANG SUN, CUI XU, KUN WANG

**Affiliations:** Department of Ultrasonic Medicine, The Affiliated Hospital of Binzhou Medical College, Binzhou, Shandong 256603, P.R. China

**Keywords:** ultrasonograph, kidney, fibrosis, acoustic radiation force impulse imaging

## Abstract

This study aimed to explore the assessment value of virtual touch quantization (VTQ) for the clinicopathological typing of renal fibrosis. The quantitative detection of 76 patients with nephropathy was performed using acoustic radiation force impulse imaging (ARFI). The extent of the renal fibrosis in each patient was confirmed using ultrasound-guided biopsy pathology. The VTQ values were compared with the degree of renal fibrosis in order to analyze the correlation between them. Patients were divided pathologically into four groups, as follows: non-fibrosis (n=14), mild fibrosis (n=40), moderate fibrosis (n=21) and severe fibrosis (n=1). Compared with the non-fibrosis group, the VTQ values of the mild and moderate fibrosis groups were significantly increased (P<0.01); however, there was no significant difference between the VTQ values of the mild and moderate fibrosis groups (P>0.05). According to the receiver operating characteristic (ROC) curve, a VTQ value of renal parenchyma of >1.67 m/sec was determined to be an indicator of renal fibrosis, with a sensitivity of 86.3% and a specificity of 83.3%. VTQ technology may be significant in the assessment of the extent of renal fibrosis.

## Introduction

The incidence of chronic kidney disease (CKD) is >7% per year, and is increasing annually. According to the latest data from the International Society of Nephrology (ISN), >500 million individuals worldwide suffer from various kidney diseases, and CKD, complicated by cardiovascular and cerebrovascular diseases, is the cause of mortality for >1 million individuals every year. CKD has become a serious threat to human health, following cardiovascular and cerebrovascular disease, cancer and diabetes. Patients with CKD are treated using lifelong dialysis or renal transplantation, putting a huge economic burden on families, medical institutions and society. Renal fibrosis is a pathophysiological process, in which the kidney function is gradually damaged. Further damage ultimately leads to a loss of function. The early treatment of renal fibrosis may delay or even reverse the fibrotic lesions; thus, the early diagnosis of renal fibrosis is necessary. The diagnosis of mild renal fibrosis is difficult, and biopsy remains the clear gold diagnostic standard. Acoustic radiation force impulse imaging (ARFI) is a novel technology that has been used in the differential diagnosis of liver fibrosis, and breast and thyroid benign and malignant tumors. The value of ARFI has been widely recognized, particularly for the diagnosis of liver fibrosis (1–4). However, its significance in the diagnosis of renal fibrosis has yet to be elucidated. The aim of the present study was to investigate the assessment value of virtual touch quantization (VTQ) in the pathological typing of renal fibrosis.

## Patients and methods

### Subjects

In total, 76 patients [43 males and 33 females; age, 11–75 years (40.37±16.13 years)], who were admitted for CKD to the Department of Nephrology Medicine, The Affiliated Hospital of Binzhou Medical College (Binzhou, China) between June 2010 and June 2012, were recruited and enrolled in this study. The extent of renal fibrosis of each patient was verified using ultrasound-guided renal biopsy pathology. This study was conducted in accordance with the Declaration of Helsinki and with approval from the Ethics Committee of Binzhou Medical College (Binzhou, China). Written informed consent was obtained from all participants.

### Methods

Experiments were performed using a Siemens Acuson S2000™ color Doppler ultrasound system (Siemens Medical Solutions USA, Inc., Detroit, MI, USA), equipped with ARFI technology software and a 4C1 probe with frequencies ranging between 2.0 and 5.0 MHz and a function of VTQ determination. The patient was asked to lie in prone position and was initially examined using conventional two-dimensional ultrasound, with a longitudinal section scanning of the right kidney. The sound beam was maintained as perpendicular as possible to the kidney capsule. Subsequently the patient was asked to hold their breath, and the VTQ sampling box was placed in the right renal subcapsular parenchyma as the image stabilized in the VTQ mode. Each position of each patient was assessed five times and the average VTQ value was recorded.

### Puncture biopsy

Puncture biopsy of the right inferior pole of the kidney parenchyma was performed using an 18G transfixion pin of a Bard automatic biopsy device (Bard Peripheral Vascular Inc., Tempe, AZ, USA) under ultrasonic guidance. A 2.2-cm kidney tissue biopsy was obtained and subsequently fixed in 10% formalin, prior to being sent for pathological examination. The patients were divided into four groups according to the extent of their kidney fibrosis, as follows: non-fibrosis, mild fibrosis (fibrosis extent, ≤25%), moderate fibrosis (fibrosis extent, 26–50%) and severe fibrosis (fibrosis extent, >50%).

### Statistical analysis

The SPSS software program version 16.0 (SPPS Inc., Chicago, IL, USA) was used for statistical analyses. Results are presented as the mean ± standard deviation. The differences between the groups were compared using an independent sample t-test. P<0.05 was considered to indicate a statistically significant difference.

## Results

**Figure 1:**
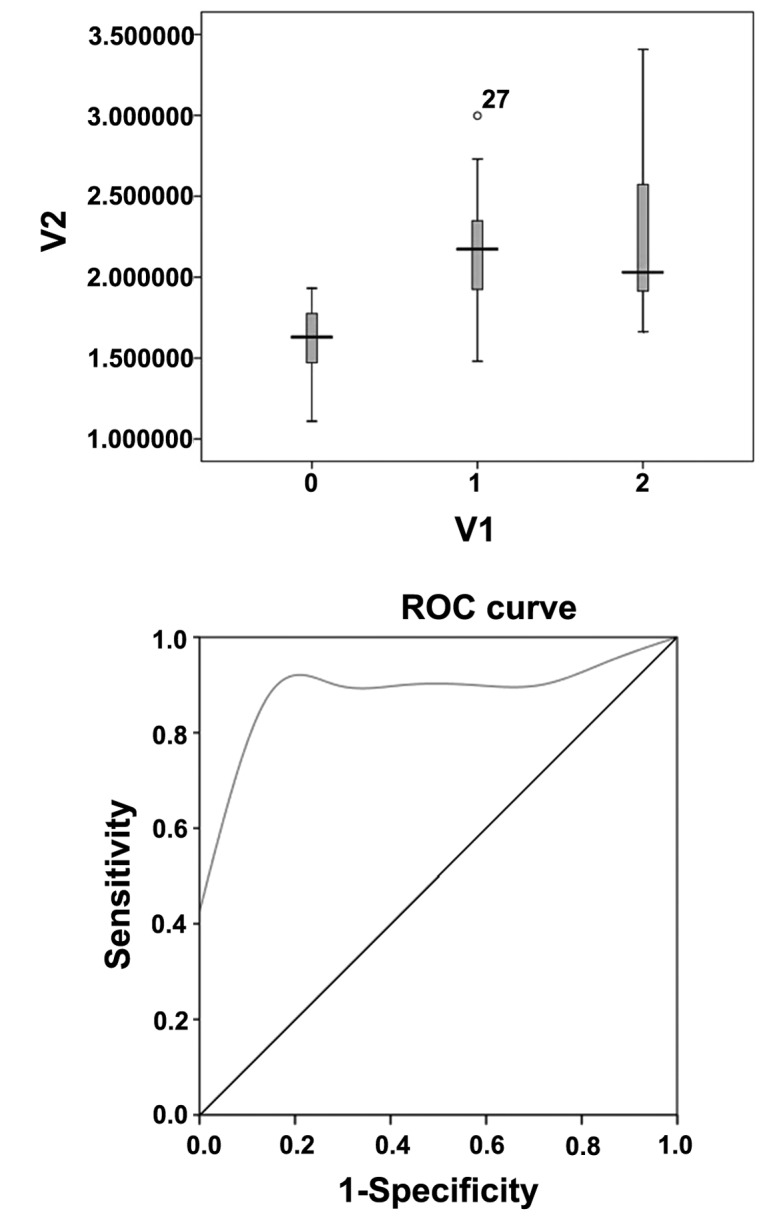
VTQ values and the ROC curve of renal parenchyma. (A) VTQ values in the non-fibrosis, mild fibrosis and moderate fibrosis groups. (B) ROC curve of the VTQ value. The VTQ value of 1.67 m/sec was determined to be a diagnostic cutoff value, with a sensitivity of 86.3% and a specificity of 83.3%. VTQ, virtual touch quantization; ROC, receiver operator curve.

The kidney tissue biopsies sent for pathological examination were classified into four groups according to the extent of fibrosis, as follows: non-fibrosis (n=14), mild fibrosis (n=40), moderate fibrosis (n=21) and severe fibrosis (n=1). The corresponding VTQ values were 1.59±0.14, 2.15±0.38, 2.29±0.53 and 2.24 m/sec, respectively (Table I). As there was only one patient in the severe fibrosis group, this group was excluded from statistical analysis. Pairwise comparisons of the non-fibrosis group with the mild and moderate fibrosis groups demonstrated that the VTQ values were significantly increased in the mild and moderate fibrosis groups (P<0.01). However, there was no significant difference between the mild and moderate fibrosis groups (P>0.05). The distribution of the VTQ values of renal parenchyma in the non-fibrosis, mild fibrosis and moderate fibrosis groups are shown in Fig. 1A. Furthermore, a receiver operating characteristic (ROC) curve was created using the VTQ values of the non-fibrosis and mild fibrosis groups. It was demonstrated that an elevated VTQ value has a certain discriminant value in the diagnosis of renal fibrosis (P<0.01, Fig. 1B). A measured VTQ value of renal parenchyma >1.67 m/sec was deemed to be a diagnostic indicator for renal fibrosis. The diagnostic sensitivity and specificity of the VTQ value were 86.3 and 83.3%, respectively.

## Discussion

The common pathway for the development of CKD, such as primary and secondary glomerular diseases, tubular, interstitial and vascular diseases, and renal transplant chronic rejection disease, to terminal nephropathy is kidney fibrosis. The main pathological change is the absence of normal renal units*,* which are replaced by a large quantity of fibroblasts and myofibroblasts. The generation and accumulation of extracellular matrix, including collagen fibers and fibronectin, causes glomerulosclerosis and tubulointerstitial fibrosis and ultimately leads to the loss of kidney function (5,6). The pathological change in renal fibrosis is a gradual evolutive process from light to severe. It may be inhibited or delayed by early medication, but in severe instances it is irreversible. Therefore, the early diagnosis of renal fibrosis is likely to be beneficial for the treatment of the disease (7). At present, the clinical diagnosis of renal fibrosis is dependent on renal biopsy. However, a needle biopsy is an invasive diagnostic method with a degree of risk involved. Thus, a new, non-invasive and repeatable diagnostic method is required.

Flexibility is an important physical property of biological tissues. The flexibility is not only different among various tissue types, but also in the pathological states of the same tissue. As a result of this, ultrasound elasticity imaging techniques have emerged. Ophir *et al*(8) first proposed the concept of elastography in 1991. Following 20 years of research and development, ultrasound elasticity imaging technology has been widely used in the differential diagnosis of benign and malignant superficial organs, such as the breast and thyroid (9–12). It has also been used in the diagnosis of liver fibrosis and cirrhosis (1–4) and in the study of various pathological types of advanced gastric cancer (13). Based on ultrasound elastography, ARFI is an established imaging technology, which includes virtual touch tissue imaging (VTI) and VTQ. VTQ is an ultrasound imaging technology for the quantitative assessment of tissue elasticity. It only produces target displacement in the region of interest, but no overall displacement, which makes up the qualitative deficiency of the past ultrasound elasticity imaging technology. VTQ is an absolute quantitative indicator that is capable of supporting tissue image contrast between patients. The ARFI technique has been shown to be capable of measuring the quantitative flexibility of abdominal organs, including the liver, kidney and stomach (13–16). A recent investigation demonstrated the application of ARFI for the study of pancreatic cystic lesions (17).

VTQ technology for the diagnosis of renal fibrosis has been rarely reported and remains controversial, due to the current lack of a unified quantitative standard. Stock *et al*(18) hypothesized that renal parenchymal was likely to harden as a result of fibrosis and that the elasticity was likely to decrease when lesions occurred in the kidney. In addition, it was hypothesized that the VTQ value or the Young’s modulus of the renal cortex were likely to have a corresponding change that was potentially meaningful for the early diagnosis of renal fibrosis. However, Syversveen *et al*(19,20) reported that VTQ was not able to significantly distinguish mild fibrosis from non-fibrosis in a transplanted kidney. Furthermore, there are great differences in the VTQ values of different examiners.

In this study, 76 patients with fibrosis, in whom the extent of the fibrosis had been confirmed using renal puncture biopsy, were analyzed using VTQ. It was observed that the VTQ values of patients with mild renal fibrosis were significantly higher than those of the non-fibrosis group (P<0.01). However, the VTQ values of patients with mild renal fibrosis showed no significant difference from the patients with moderate renal fibrosis. The severe fibrosis group was excluded as there was only one patient classified as having severe fibrosis, which was insufficient for a statistical analysis. According to the maximum area under the ROC curve, a VTQ value of >1.67 m/sec was determined as a diagnostic indicator of mild fibrosis of the renal cortex with a sensitivity of 86.3% and a specificity of 83.3%. The VTQ technique provides a novel reference for the clinical diagnosis of renal fibrosis. This is consistent with our assumption and results of the study of Stock *et al*(18). However, there are certain limitations in this study, the VTQ technique is not capable of accurately differentiating between light and moderate renal fibrisis. This may be due to the fact that there are large numbers of cross-data between light and moderate renal fibrosis. Furthermore, the classification in the present study was not entirely based on the differences between light and moderate renal fibrosis. The VTQ value measured in the non-fibrosis nephropathy group demonstrated a certain degree of overlap with that of the mild fibrosis group and the moderate fibrosis group. As the sample sizes in this study were small, particularly for the severe renal fibrosis group, further studies with a larger sample size and more data are required in order to confirm the findings of this study.

## Figures and Tables

**Table I:** Case numbers and VTQ values of the four groups.

Groups	Case number	VTQ value
Non-fibrosis	14	1.59±0.14
Mild fibrosis	40	2.15±0.38^a^
Moderate fibrosis	21	2.29±0.53^a^
Severe fibrosis	1	2.24

Data of VTQ values are presented as the mean ± standard deviation.

a P<0.01 vs. non-fibrosis group.

VTQ, virtual touch quantization.
